# Treatment and survival of patients with pancreatic ductal adenocarcinoma: 15-year national cohort

**DOI:** 10.1093/bjsopen/zrac004

**Published:** 2022-03-08

**Authors:** Linn Såve Nymo, Tor Åge Myklebust, Hanne Hamre, Bjørn Møller, Kristoffer Lassen

**Affiliations:** 1 Department of Gastrointestinal Surgery, University Hospital of North Norway, Tromsø, Norway; 2 Institute of Clinical Medicine, Arctic University of Norway, Tromsø, Norway; 3 Department of Clinical and Registry-based Research, Cancer Registry of Norway, Oslo, Norway; 4 Department of Oncology, Akershus University Hospital, Lørenskog, Norway; 5 Department of Hepatobiliary and Pancreatic Surgery, Oslo University Hospital Rikshospitalet, Oslo, Norway

## Abstract

**Background:**

Improvement in survival from pancreatic ductal adenocarcinoma (PDAC) has been reported in trial settings but is less explored in unselected cohorts. The aim of this study was to assess trends in provision of treatments and survival in Norway over a 15-year period following the implementation of hepato-pancreato-biliary (HPB) multidisciplinary teams, centralization of surgery, and implementation of modern chemotherapy (CTx) regimens.

**Methods:**

A population-based observational study was conducted by analysing all patients diagnosed with PDAC between 2004 and 2018 using coupled data from the Cancer Registry of Norway and the National Patient Registry.

**Results:**

A total of 10 630 patients were identified, of whom 1492 (14.0 per cent) underwent surgical resection. The resection rate, median age of those resected, and provision of perioperative CTx all increased over time. Median overall survival after resection improved from 16.0 months in the period 2004 to 2008 to 25.1 months in the period 2014 to 2018 (*P* < 0.001). For non-resected patients there was a rise in the provision of palliative chemotherapy, but little survival gain over time (median overall survival for 2004 to 2008 was 3.2 months *versus* 4.2 months for 2014 to 2018; *P* < 0.001). The rate of patients who did not receive any tumour-directed treatment (neither CTx nor surgery) was 44.3 per cent (2481 of 5603 patients) and decreased from 52.9 per cent in 2010 to 37.9 per cent in 2018 (*P* < 0.001). The median overall survival for all patients with PDAC increased from 3.7 months for 2004 to 2008 to 5.8 months for 2014 to 2018 (*P* < 0.001).

**Conclusion:**

Survival after resection increased substantially, as did national resection rates. Little development in the provision of CTx or survival was observed for non-resected patients.

## Teaser

A complete 15-year (2004-2018) national cohort of patients with pancreatic ductal adenocarcinoma was analyzed for trends in tumour-directed treatment and survival. The resection rates, age among resected and survival after resection increased and more patients received perioperative chemotherapy. For non-resected patients there was little change in provision of chemotherapy and survival, and still 4 in 10 patients receive no tumor-directed treatment.

## Introduction

Pancreatic ductal adenocarcinoma (PDAC) remains one of the most lethal malignancies^1,2^. Long-term survival is still almost an oddity, even when the tumour is resected^3,4^. The majority of patients present at either an inoperable disease stage or with advanced age or other frailty barring them from full tumour-directed treatment^5^.

Substantial efforts have been devoted to improving the survival rate from PDAC. Among them, vascular resection techniques^6^ and neoadjuvant chemo- (CTx) and radiotherapy have been increasingly adopted to expand resectability criteria^7–9^. The FOLFIRINOX regimen (oxaliplatin, irinotecan, fluorouracil, and leucovorin) has superseded gemcitabine-based regimens at the expense of increased toxicity^10^. Novel immunotherapy-based treatment options and treatments based on molecular tumour analysis are also emerging but so far with disappointing results^11,12^. Although moderate increases in median survival among patients eligible for treatment have been observed, improvement in long-term survival is still awaited, in unselected populations.

The population of Norway, currently around 5.4 million, is served by a public healthcare system with universal governmental coverage. No private alternatives for cancer treatment exist. The nation is divided into four independent regional health authorities. As a result of a gradual centralization process during the past two decades, pancreatic resections are now performed in only one hepato-pancreato-biliary (HPB) unit in each region (two units in the western region), and HPB unit multidisciplinary team decisions on resectability have become mandatory. CTx is instituted and monitored at dedicated oncology units and usually completed in an outpatient, decentralized setting. FOLFIRINOX has been used as palliative regimen since 2012 and in a perioperative setting since 2018.

The main body of evidence documenting improvements in PDAC treatment reflects what may be accomplished for the small minority of patients who were amenable to full multimodal treatment, including extensive surgery and/or CTx regimens with high toxicity, and who have received treatment in expert centres or within trial settings. Whether the improvements documented for these few are detectable in large, unselected population-based cohorts is largely unknown. This study aimed to investigate the trends in tumour-directed treatment for PDAC in Norway to assess whether the logistical, surgical, and oncological developments of the latter 15 years have resulted in improved survival.

## Methods

### Ethics

Access to data and analyses were approved by separate applications to the Regional Research Ethics Committee (REK Nord, reference no. 81594), the Data Protection Office at the Arctic University of Norway (UiT, Tromsø), Cancer Registry of Norway (CRN), and the Norwegian Health Directory.

### Study design

This study was an observational cohort study including all patients diagnosed with ductal adenocarcinoma of the pancreas (PDAC) between 2004 and 2018 in Norway. The STROBE guidelines for reporting observational studies were adhered to^13^. Patients were identified within the CRN database and the basis for the PDAC diagnosis was registered. The CRN dataset is linked to the Norwegian Cause of Death Registry, the National Population Registry (vital status), and the National Patient Registry (NPR). Data on both resection type and provision and setting of CTx (neoadjuvant, adjuvant or palliative) were available only for the last 9 years of the cohort (2010 to 2018) as the NPR data were incomplete before 2010 (*Fig. S1*).

### Data collection and quality

CRN, NPR, the National Cause of Death Registry, and the National Population Registry are population registries with automatic data accrual and are all considered complete and with high-quality data^14^. CRN combines several data sources to identify patients, including clinical notifications, pathology reports (cytology, biopsies, surgical specimens, and autopsies), and death certificates. Morphology codes (ICD3-O) are provided in *Table S1*. Data on the provision and timing of CTx were gathered through a combination of procedure codes (primarily Nomesco Classification of Medical Procedures code WBOC*) and ICD-10 diagnostic codes (Z51.1) registered within the NPR.

### Definitions

#### Resection rate

Resection rate was defined as the rate among all patients with a PDAC diagnosis (all stages) who underwent tumour resection.

#### CTx

Neoadjuvant CTx was defined as any CTx given within the last 12 weeks prior to pancreatic resection of any type. Adjuvant CTx was defined as CTx initiated within 12 weeks after resection. Palliative CTx was defined as CTx given between date of diagnosis and death for non-resected patients; CTx commenced not earlier than 6 months after surgery for resected patients who did not undergo adjuvant CTx; or CTx commenced later than 9 months after surgery for resected patients who did receive adjuvant CTx.

Preoperative CTx for borderline resectable disease was, in line with the International Study Group of Pancreatic Surgery (ISGPS)^15^, assigned by the term neoadjuvant, although some controversies on the use of this term for borderline resectable disease exist. The study design did not allow for identification of the small subset of patients with locally advanced disease who were downstaged by CTx and reached resection. These patients were also grouped as receiving neoadjuvant CTx (*Fig. S2*).

Radiotherapy was rarely applied in both a perioperative and palliative setting in Norwegian patients and therefore not included in the analyses.

#### Incidence and survival

All registered PDAC diagnoses were included in the calculation of incidence rates, but patients notified only through death certificates or autopsies were excluded from analyses of survival and provision of treatment. Survival was assessed as time from PDAC diagnosis to death, emigration, or end of follow-up (31 December 2019), whichever came first.

### Statistics

Demographic data are reported as medians and interquartile ranges (i.q.r.) or means with standard deviation (s.d.), as fit. Trend analyses were done by linear or logistic regression, with time (calendar year) treated as a continuous variable. Resection rates and use of CTx were compared by χ^2^ tests with effect measures reported as odds ratios. Survival curves were estimated by the Kaplan–Meier method and compared using log-rank tests. Multivariable Cox proportional hazard regression models were estimated to present age-adjusted hazard ratios of death (all causes) across time periods. Provision of CTx and surgery were treated as time-varying covariates in all survival analyses in order to avoid immortal time bias. Owing to the short follow-up time, patients diagnosed in 2018 were omitted when calculating simple proportions of palliative CTx used for trend analyses (but included in survival analyses as time-varying covariates). Length of follow-up was calculated as simple median follow-up times of all individuals from diagnosis to death or loss to follow-up.

The level of statistical significance was set at *P* = 0.05, and the provided confidence intervals (c.i.) were at 95 per cent. STATA version 16.1 (StataCorp, College Station, Texas, USA) and SPSS Statistics version 26.0 (IBM, Armonk, New York, USA) were used for statistical analyses.

## Results

A total of 10 630 patients were diagnosed with PDAC, of whom 1492 (14.0 per cent) and 9138 (86.0 per cent) did and did not undergo resection of the primary tumour, respectively (*Table 1*).

**Table 1:** Patient demographics 2004–2018

Patient demographics	Resected (*n* = 1492)	Non-resected (*n* = 9138)
**Male sex**	780 (52.3)	4437 (48.6)
**Age, median (IQR)**	68 (60–74)	75 (66–83)
**Distribution, age groups**		
< 60	347 (23.3)	1114 (12.2)
60–74	807 (54.1)	3429 (37.5)
75+	338 (22.7)	4595 (50.3)

Data are *n* (%) or median (i.q.r.).

### Data completeness and follow-up time

The number of patients who could not be traced through the registries for 2004 to 2008, 2009 to 2013, and 2014 to 2018 was one, three, and two patients, respectively. A total of 29 (2004 to 2008), 79 (2009 to 2013), and 352 (2014 to 2018) patients were administratively censored by time of data retrieval for being alive. The median follow-up time for the complete cohort was 4.4 months, with stratified numbers for resected and non-resected patients of 19.1 months and 3.3 months, respectively.

### Demographics (2004 to 2018)

The incidence of PDAC in Norway was 14.3 per 100 000 inhabitants for the whole study period. There was a significant increase in incidence during the study period (*P* < 0.001) and the stratified rates per 100 000 were 14.1, 13.8, and 15.0 for 2004 to 2008, 2009 to 2013, and 2014 to 2018, respectively.

The rate of diagnosis verification by cytology/biopsy in non-resected patients rose from 59.9 per cent in 2004 to 74.1 per cent in 2018 (*P* < 0.001), and the proportion of non-resected patients who were first diagnosed with PDAC at death certificate and/or autopsy for the whole study period was 395 of 9138 (4.3 per cent).

Specific procedure codes in the NPR^16^ were only available from 2010 to 2018. Of the 1045 patients who were resected in this period, a procedure code was available for 974 patients (93.2 per cent). Of these, 710 (72.9 per cent) underwent pancreatoduodenectomy (JLC 30/31), 199 (20.4 per cent) distal resection (JLC 10/11), 62 (6.4 per cent) total pancreatectomy/total pancreatoduodenectomy (JLC 20/21/40/41), and three patients (0.3 per cent) other type of resection. Ninety-day mortality after resection occurred in 46 of 1492 patients (3.2 per cent) and no change was observed during the study period (*P* = 0.161). The median age of resected patients increased from 66 (i.q.r. 58–74) years in 2004 to 70 (i.q.r. 62–75) years in 2018 (*P* < 0.001).

### Resection rates

**Fig. 1:**
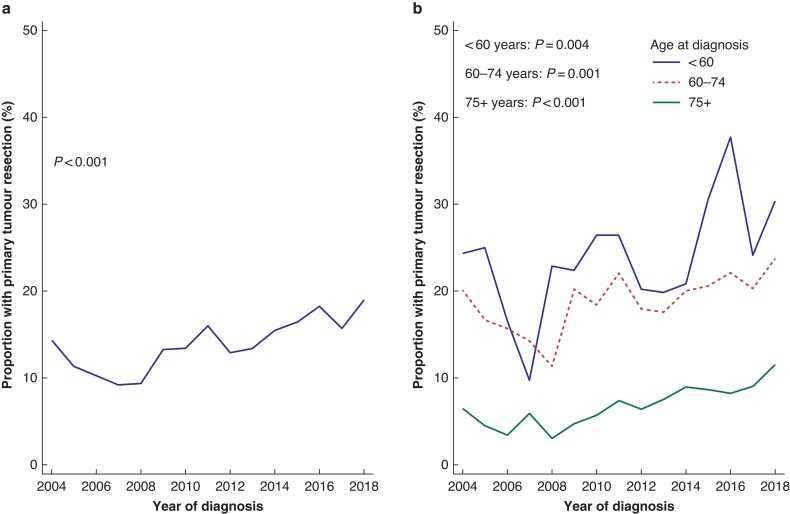
Trend in resection rate 2004–2018 Trend in resection rate for **a** all ages and **b** stratified by age group.

The resection rate increased during the study period, from 355 of 3287 (10.8 per cent) for 2004 to 2008 up to 667 of 3925 (17.0 per cent) for 2014 to 2018, and peaked for 2018 (18.9 per cent) (*Fig. 1a*). The age-adjusted overall odds ratio for resection in 2014 to 2018 was 1.54 (95 per cent c.i. 1.15 to 2.05), with 2004 to 2008 as the reference. The increase in resection rate was significant for all three age groups (*Fig. 1b*). With 2004 to 2008 as the reference, the odds ratio for resection in 2014 to 2018 was 1.62 (95 per cent c.i. 1.21 to 2.17) for patients aged less than 60 years, 1.49 (95 per cent c.i. 1.22 to 1.81) for patients aged 60 to 74 years, and 2.11 (95 per cent c.i. 1.59 to 2.79) for patients aged 75 years or older. There was no difference in total resection rates for 2004 to 2018 between the populations of the four regional health authorities (range 13.1 to 14.3 per cent; *P* = 0.807).

### Provision of chemotherapy (2010 to 2018)

**Fig. 2:**
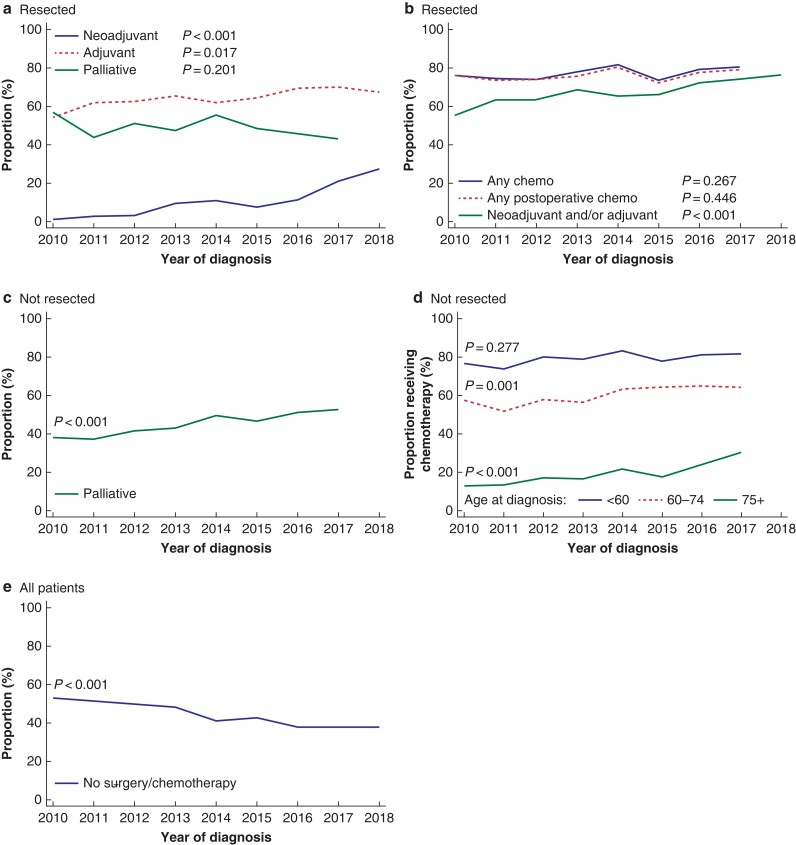
Trend in provision rate of tumour-directed treatment **a** Neoadjuvant, adjuvant, and palliative chemotherapy among resected patients (non-distinct curves); **b** any chemotherapy (all settings), any perioperative and any postoperative chemotherapy for resected patients (non-distinct curves); **c** palliative chemotherapy for non-resected patients (all ages); **d** palliative chemotherapy for non-resected patients stratified by age; **e** proportion of patients diagnosed with pancreatic ductal adenocarcinoma who did not receive any tumour-directed treatment (resection and/or chemotherapy).

Data for the resected population since 2010 are shown in *Fig. 2a,b*. In total 125 of 1045 patients (12.0 per cent) received neoadjuvant CTx and there was a marked increase over time from 1.2 per cent to 27.4 per cent between 2010 and 2018 (*P* < 0.001). The odds ratio for receiving neoadjuvant CTx was for patients diagnosed between 2014 and 2018 was 4.44 (95 per cent c.i. 2.58 to 7.63) *versus* 2010 to 2013. A total of 676 of 1045 (64.7 per cent) patients commenced adjuvant CTx. The rate of administration of adjuvant CTx increased over time from 54.2 per cent in 2010 to 67.3 per cent in 2018 (*P* = 0.017). The odds ratio for receiving adjuvant CTx in 2014 to 2018 was 1.27 (95 per cent c.i. 0.98 to 1.65) *versus* 2010 to 2013. Combined, 712 of 1045 resected patients (68.1 per cent) were provided with perioperative (any) CTx, and the rate increased from 55.4 per cent in 2010 to 76.2 per cent in 2018 (*P* < 0.001). The odds ratio for being provided with perioperative CTx was 1.46 (95 per cent c.i. 1.12 to 1.91) in 2014 to 2018 *versus* 2010 to 2013. In total, 475 patients (45.5 per cent) received palliative CTx after surgery without significant differences during the study period.

Among resected patients who did not receive adjuvant CTx, 111 of 369 (30.1 per cent) commenced palliative CTx and among those who started adjuvant CTx 364 of 676 (53.9 per cent) received palliative CTx. A total of 787 of 1045 (75.3 per cent) resected patients were provided with postoperative CTx (adjuvant and/or palliative) within 12 weeks after resection and up to their day of death, and this did not change over time (*P* = 0.446). The overall rate of provision of CTx (any setting) to resected patients did not change during the study period (*P* = 0.276).

Data for patients who did not undergo resection are shown in *Fig. 2c–e*.

Altogether, 1813 of 4013 patients (45.0 per cent) commenced on palliative CTx between 2010 and 2017. Non-resected patients (all ages) diagnosed between 2014 and 2017 had an odds ratio of receiving palliative CTx of 1.49 (95 per cent c.i. 1.32 to 1.69) *versus* patients diagnosed in 2010 to 2013. In subgroup analyses stratified by age, the increase was significant in the two oldest age groups with an odds ratio of palliative CTx for 2014 to 2017 for patients aged younger than 60 years of 1.26 (95 per cent c.i. 0.83 to 1.93), for patients aged 60 to 74 years of 1.42 (95 per cent c.i. 1.17 to 1.72), and for those aged 75 or older of 1.72 (95 per cent c.i. 1.35 to 2.20), with 2010 to 2013 as the reference.

The proportion of patients diagnosed between 2010 and 2018 who did not receive any tumour-directed treatment (neither CTx nor surgery) was 2481 of 5603 (44.3 per cent), which decreased from 52.9 per cent in 2010 to 37.9 per cent in 2018 (*P* < 0.001).

### Overall survival

**Fig. 3:**
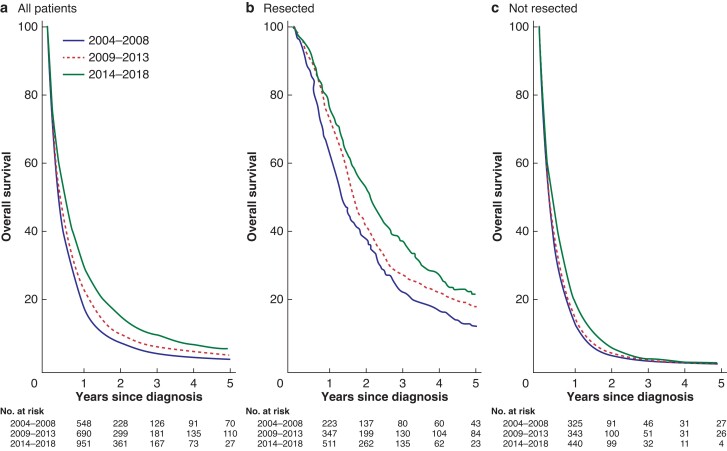
Kaplan–Meier survival plots **a** All patients; **b** resected patients; and **c** non-resected patients with stratified curves for the respective time cohorts. Numbers at risk provided in tables under each figure.

Overall survival and hazard ratios for death over time are shown in *Table 2a–d* and *Fig. 3*.

**Table 2:** Overall survival after diagnosis for (a) all patients diagnosed with pancreatic ductal adenocarcinoma (PDAC), (b) all resected patients, (c) all non-resected patients, and (d) non-resected patients stratified by provision of chemotherapy (2010 to 2018 data only)

a Overall survival for all patients diagnosed with PDAC 2004–2018 (*n* = 10 630)			
Time period	Median (i.q.r.) months	1-year, % (95% c.i.)	HR of death (95% c.i.)
**2004–2008**	3.7 (1.4–9.1)	17.8 (16.4–19.1)	Ref.
**2009–2013**	4.2 (1.5–11.0)	22.8 (21.3–24.3)	0.89 (0.85–0.92)
**2014–2018**	5.8 (1.7–14.7)	29.9 (28.4–31.5)	0.73 (0.70–0.76)

b Overall survival for patients resected for PDAC 2004–2018 (*n* = 1492)								
Time period	Median (i.q.r.) months	1-year, % (95% c.i.)	3-year, % (95% c.i.)	5-year, % (95% c.i.)	HR of death (c.i.)			
					All ages	< 60	60–74	75+
**2004–2008**	16.0 (8.3–33.0)	62.3 (57.0–67.1)	22.0 (17.9–26.4)	11.8 (8.8–15.4)	Ref.	Ref.	Ref.	Ref.
**2009–2013**	19.8 (11.2–40.1)	72.8 (68.5–76.7)	27.2 (23.3–31.2)	17.6 (14.3–21.1)	0.81 (0.70–0.93)	0.75 (0.55–1.00)	0.83 (0.67–1.01)	0.83 (0.61–1.12)
**2014–2018**	25.1 (12.4–49.2)	75.9 (72.3–79.0)	36.4 (32.2–40.6)	21.5 (17.2–26.3)	0.65 (0.57–0.76)	0.64 (0.47–0.87)	0.69 (0.56–0.85)	0.59 (0.43–0.79)

c Overall survival for non-resected patients diagnosed with PDAC 2004–2018 (*n* = 9138)			
Time period	Median (i.q.r.) months	1-year, % (95% c.i.)	HR of death (95% c.i.)
**2004–2008**	3.2 (1.3–7.4)	17.8 (16.4–19.1)	Ref.
**2009–2013**	3.3 (1.3–7.8)	22.8 (21.3–24.3)	0.97 (0.92–1.03)
**2014–2018**	4.2 (1.5–9.8)	29.9 (28.4–31.5)	0.86 (0.81–0.90)

d Overall survival for non-resected patients stratified by provision of palliative chemotherapy 2010–2018 (*n* = 4013)				
Time period	Chemotherapy		No chemotherapy	
	Median (i.q.r.), months	HR of death (95% c.i.)	Median (i.q.r.), months	HR of death (c.i.)
**2010–2013**	6.5 (3.3–11.3)	Ref.	2.3 (1.2–5.1)	Ref.
**2014–2018**	7.2 (3.5–12.6)	0.90 (0.82–0.98)	2.6 (1.3–6.4)	0.90 (0.83–0.97)

HR, hazard ratio.

There was no difference in overall survival between the regions, considering entire regional complete PDAC populations (*P* = 0.063) or resected patients only (*P* = 0.536).

## Discussion

**Fig. 4:**
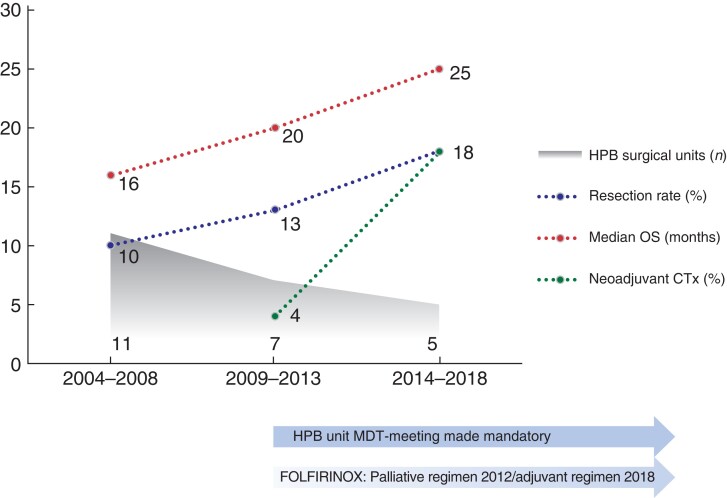
Concurrent development during the study period for HPB service organization and core treatment metrics and outcomes for resectable pancreatic adenocarcinoma

The current study provides information on the treatment and survival of patients with PDAC in an everyday setting in a public healthcare system. This 15-year cohort captured the processes of establishing HPB unit decision-making on resectability and centralization of surgery, along with the widespread use of extended surgery (e.g. vascular resection) and modern oncological care. During the study period, an increasing proportion of patients received tumour-directed treatment and a subsequent survival benefit over time was observed in patients amenable to therapy (*Fig. 4*). Despite this, around 40 per cent of patients did not receive surgery or CTx and succumbed within a few months of being diagnosed, which heavily influenced the poor overall survival for the entire population.

Resected patients had the largest observed survival gain throughout the study period, with an improvement in median survival of nearly one full year and in overall 3-year survival up to 36 per cent (*Table 2*). These data are in line with^17,18^ or somewhat better than^19^ other national or population-based cohort studies, but still inferior to selected resection series^20^ or landmark randomized controlled trials^10,21^. The improved survival after resection was accompanied by a 54 per cent increase in resection rate, which notably excludes stricter criteria for resection as a potential cause of bias. A similar upsurge in resection rates after the centralization of pancreatic cancer surgery has also been documented in the Netherlands^22^. In the current study, the highest increase in resection rate was found in the oldest age group, and the median age among those resected increased by 4 years during the study period. The 90-day mortality rate after pancreatic resection (3.2 per cent) was low compared to contemporary studies^23–25^, and this probably weighs on the threshold to perform surgery in the elderly and frail with early-stage disease. Of note, the 90-day mortality did not decrease during the study time frame, so it did not influence the improved long-term survival.

Centralization of surgery and regional HPB multidisciplinary discussion on resectability became mandatory during the study period, safeguarding patients’ access to advanced radiological, surgical, and oncological care across Norway. While surgical or oncological progress may have contributed to more and older patients reaching resection, a quality improvement in radiology during the decision-making process may have resulted in better selection of patients for surgery and could serve as a part of the explanation behind the increased survival post-resection^26,27^.

The Norwegian national guidelines currently recommend adjuvant CTx for all resected patients, but neoadjuvant only for patients with borderline resectable disease. The increasing use of perioperative CTx might have impacted on the overall increased survival post resection in several ways. Patients with borderline resectable tumours who either had disease progression or deteriorated during neoadjuvant CTx and never reached resection were within the dataset categorized as non-resected. Conversely, those who proceeded to resection would conceivably have had a favourable tumour biology as they passed the ‘test of time’ and this biological selection might have influenced on the survival of the resected cohort as a whole. Furthermore, provision of CTx earlier in the disease course, when patients are fitter and motivated to undergo treatment and before metastasized disease occurs, might have contributed to more complete and full-dosed CTx cycles.

Between 2010 and 2017, almost four in 10 non-resected patients started palliative CTx in Norway, in contrast to the rate of 18 per cent reported from a population study from the Netherlands^19^. The modest improvement in the administration rate and survival following palliative CTx for non-resected patients might be explained by the fact that patients were potentially less fit and with more advanced disease, given the concurrent broadening in the indications for resection and use of neoadjuvant CTx.

This study has some limitations. Subgroup analyses on specific CTx regimens or completion of CTx were not possible. Data on patients who received CTx in a neoadjuvant setting and did not subsequently undergo resection were not sufficiently detailed to allow subgroup analysis. Data on tumour stage, rate of explorative laparotomies, comorbidity, and performance status are not accessible through the CRN nor the NPR. The increased use and improvement of radiological imaging during the study time frame might have led to diagnosis at an earlier disease stage in patients in the latest study period. This opens the possibility of a lead time bias that would influence on both resection rates and survival. In addition, caution must be taken when comparing data from different cancer registries as the foundation of the datasets in terms of inclusion criteria for the diagnosis may differ.

*Disclosure*. The authors declare no conflict of interest.

## Supplementary Material

zrac004_Supplementary_Data
