# Supplementary material for: Treatment and survival of patients with pancreatic ductal adenocarcinoma: 15-year national cohort
Source: BJS Open. 2022 Mar 8;6(2):zrac004. doi: 10.1093/bjsopen/zrac004 (PMC8902330; doi:10.1093/bjsopen/zrac004)
Supplement: zrac004_Supplementary_Data [file zrac004_supplementary_data.zip › Supplementary_Table_1.docx]

**Table S1.** ICD-3-O Morphology codes

morf4 | Freq. Percent Cum.

------------+-----------------------------------

8140 | 5,314 49.99 49.99

6999 | 3,560 33.49 83.48

8500 | 814 7.66 91.14

8010 | 197 1.85 92.99

8480 | 120 1.13 94.12

8481 | 115 1.08 95.20

6900 | 102 0.96 96.16

8000 | 62 0.58 96.75

8001 | 58 0.55 97.29

8020 | 57 0.54 97.83

8453 | 39 0.37 98.19

8560 | 29 0.27 98.47

8550 | 21 0.20 98.66

8452 | 18 0.17 98.83

8470 | 17 0.16 98.99

8070 | 13 0.12 99.12

8144 | 12 0.11 99.23

8574 | 10 0.09 99.32

8490 | 9 0.08 99.41

8260 | 8 0.08 99.48

8310 | 8 0.08 99.56

8148 | 6 0.06 99.61

8012 | 5 0.05 99.66

8021 | 4 0.04 99.70

8154 | 4 0.04 99.74

8440 | 4 0.04 99.77

8211 | 3 0.03 99.80

8800 | 3 0.03 99.83

8255 | 2 0.02 99.85

8471 | 2 0.02 99.87

8523 | 2 0.02 99.89

8890 | 2 0.02 99.91

8936 | 2 0.02 99.92

8030 | 1 0.01 99.93

8035 | 1 0.01 99.94

8430 | 1 0.01 99.95

8510 | 1 0.01 99.96

8570 | 1 0.01 99.97

8572 | 1 0.01 99.98

8804 | 1 0.01 99.99

8850 | 1 0.01 100.00

------------+-----------------------------------

Total | 10,630 100.00
