# Supplementary figures and images for: Treatment and survival of patients with pancreatic ductal adenocarcinoma: 15-year national cohort
Source: BJS Open. 2022 Mar 8;6(2):zrac004. doi: 10.1093/bjsopen/zrac004 (PMC8902330; doi:10.1093/bjsopen/zrac004)

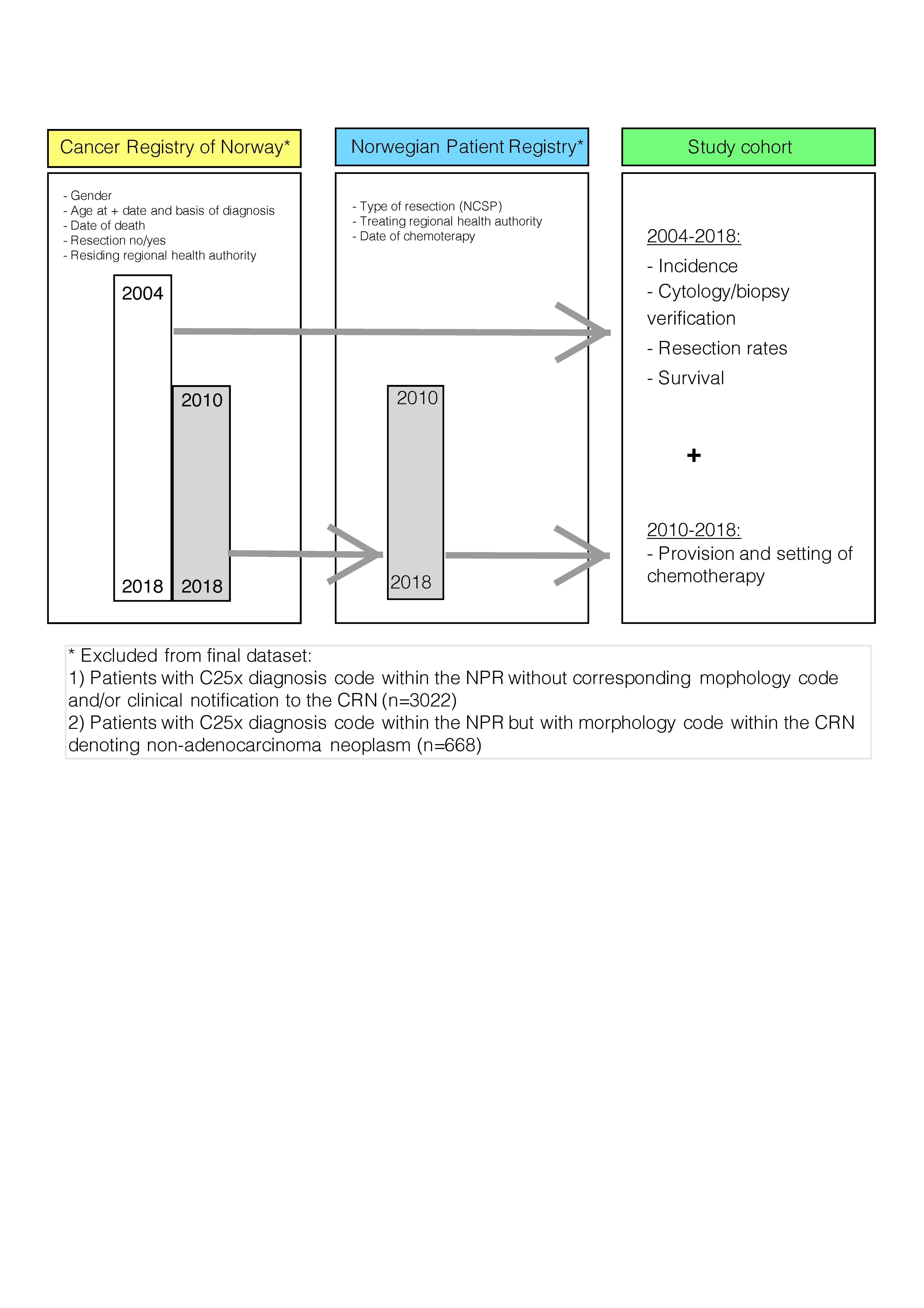

Supplement: zrac004_Supplementary_Data [file zrac004_supplementary_data.zip › Supplementary_Figure_1.jpg]

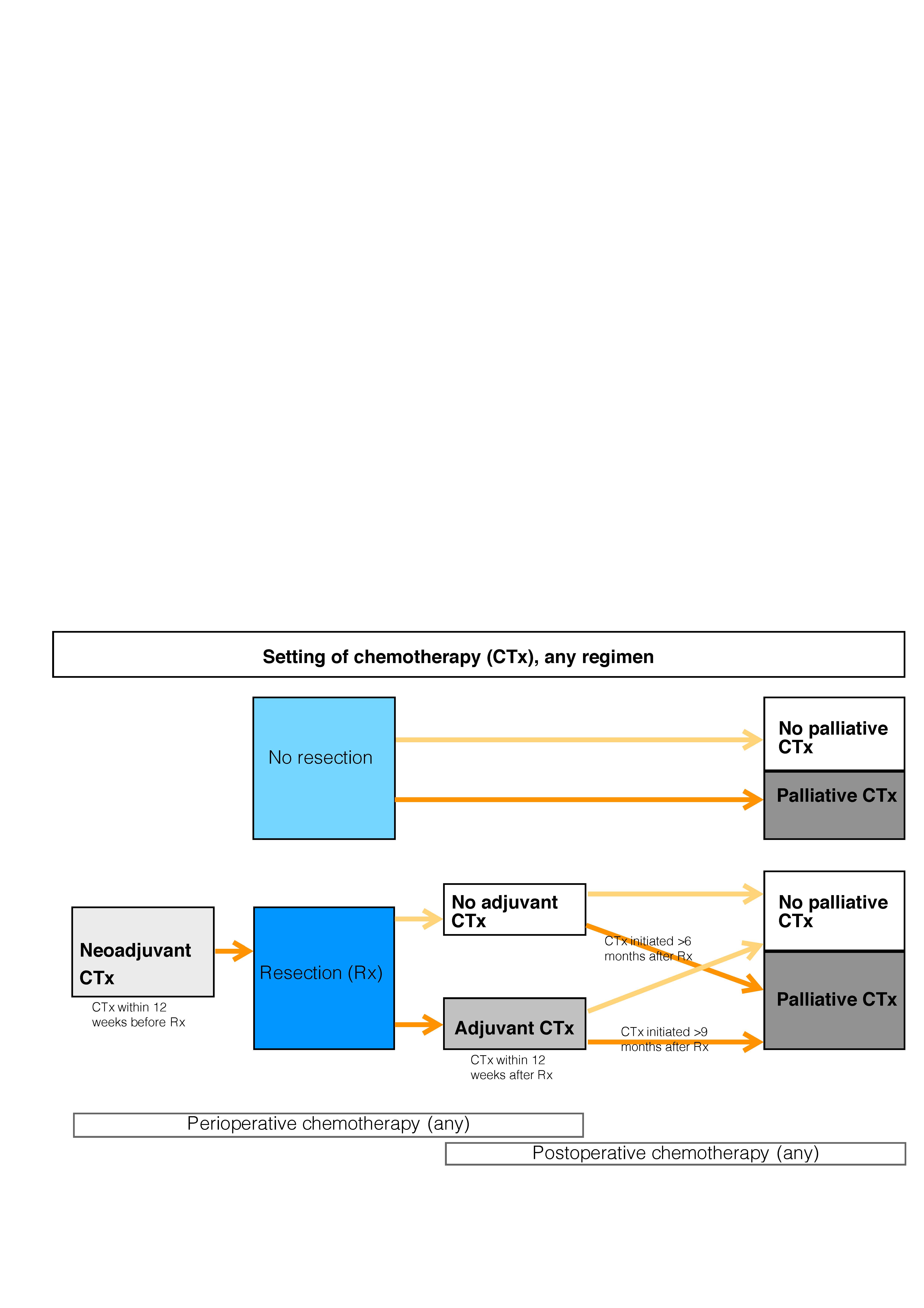

Supplement: zrac004_Supplementary_Data [file zrac004_supplementary_data.zip › Supplementary_Figure_2.jpg]
